# Ameliorating Chronic Kidney Disease Using a Whole Food Plant-Based Diet

**DOI:** 10.3390/nu12041007

**Published:** 2020-04-06

**Authors:** Kathleen E. Adair, Rodney G. Bowden

**Affiliations:** 1 Department of Health, Human Performance and Recreation, Baylor University, One Bear Place #97303 Waco, TX 76798-7303, USA; 2 Robbins College of Health and Human Sciences, Baylor University, One Bear Place #97303 Waco, TX 76798-7303, USA; Rodney_Bowden@baylor.edu

**Keywords:** whole food plant-based diet, chronic kidney disease, potential renal acid load, cardiovascular disease

## Abstract

Novel approaches to ameliorating chronic kidney disease (CKD) are warranted as most patients are undiagnosed until they begin displaying symptoms of kidney failure. There is increasing evidence that a whole food plant-based (WFPB) diet may offer benefits that slow the progression of CKD, decrease the incidence cardiovascular disease, decrease rates of diabetes and obesity, and reduce inflammation and cholesterol, which in turn can delay kidney failure and the initiation of dialysis. While animal-based protein ingestion promotes an acidic environment, inflammation and renal hyperfiltration, study authors report plant-based protein can be alkaline-producing and anti-inflammatory and can contain renoprotective properties. Although there may be benefits to adopting a WFPB diet, macronutrient and micronutrient content should be carefully considered and adjusted to avoid malnutrition in CKD patients. Further research needs to be done in order to establish the biological plausibility and feasibility of a WFPB in individuals with diagnosed CKD.

## 1. Introduction

Chronic kidney disease (CKD) is one of the top ten leading causes of premature mortality in the United States (U.S.), and its incidence is increasing [1,2]. A staggering 96% of individuals with mild to moderate decreases in kidney function and 48% of individuals with severely decreased kidney function go undiagnosed [3]. Table 1 outlines the stages of CKD as estimated by glomerular filtration rate (GFR) category. CKD is often accompanied by comorbidities that put individuals at increased risk of mortality, making it a costly and burdensome public health issue [4,5,6,7]. Beyond the medical burden, the individual consequences of developing CKD present many challenges, including a reduced quality of life for both CKD patients and their loved ones.

Risk factors for the development of CKD include type II diabetes (TIIDM), hypertension (HTN), cardiovascular disease (CVD), a family history of CKD, obesity and metabolic acidosis or metabolic syndrome [4,8,9,10,11,12,13,14,15,16,17,18]. A majority of CKD patients have HTN, and between 20% and 40% of individuals with TIIDM will go on to develop CKD [15,16,17,18,19]. The risk of all-cause mortality, including mortality due to CVD, is higher in individuals with CKD, making this disease a topic of priority in the effort to mitigate preventable death and disease [4,8].

The current treatments for individuals with CKD include blood pressure medications such as angiotensin-converting enzyme (ACE) inhibitors, cholesterol lowering medications such as statins, erythropoietin (EPO) supplements, diuretics, phosphate and/or potassium binding drugs, calcium and vitamin D supplements and a low protein renal diet. Typically, CKD patients will be placed on a renal diet that focuses on reducing individual macronutrient and micronutrient food items such as protein, potassium, phosphorus and sodium, which can slow CKD progression and symptoms of the disease [20]. However, the renal diet has not proven effective in preventing the comorbidities that accompany CKD [21]. Individuals prescribed renal diets often reduce their intake of healthier foods (i.e., leafy green vegetables, strawberries, bananas and oranges) in order to prevent the overconsumption of a particular micronutrient (i.e., potassium). Rather than focus on the macronutrient and micronutrient content of individual food items through restriction and medication, there is a need for a focus on a dietary pattern that can improve overall health in CKD patients [22]. The China Study conducted by T. Colin Campbell et al. [23] was identified as the most comprehensive study of nutrition conducted to date. This study reported a pattern of eating that reduces CVD and TIIDM risk factors and comorbidities of CKD through a whole food plant-based (WFPB) diet. This dietary pattern, similar to a vegan diet, focuses on plant products, while eliminating or minimizing all animal products including meat, fish, eggs and dairy. A WFPB diet is more restrictive than vegan or vegetarian diets, and it derives most of its caloric energy from whole, unprocessed or minimally processed carbohydrates (CHO). While the macronutrient composition can vary, this dietary pattern is typically composed of 7–15% fat, 75–80% CHO and 10–15% protein [24,25,26]. The WFPB diet further excludes from the diet highly processed and refined foods, such as isolated vegetable oils, sugars and bleached flours, which vegan and vegetarian diets do not typically prohibit. In a WFPB diet, there is a focus on fiber-filled foods that are naturally rich in vitamins and minerals, and lower in protein content and caloric density. The foods promoted in a WFPB diet include whole grains, seeds, nuts, legumes, fruits, vegetables, tubers and starchy vegetables. Evidence for the benefit of a WFPB diet has been widely documented, and it is a novel and cost-effective intervention for patients with CKD and comorbid diseases [22,23,25,26,27,28].

**Figure 1:**
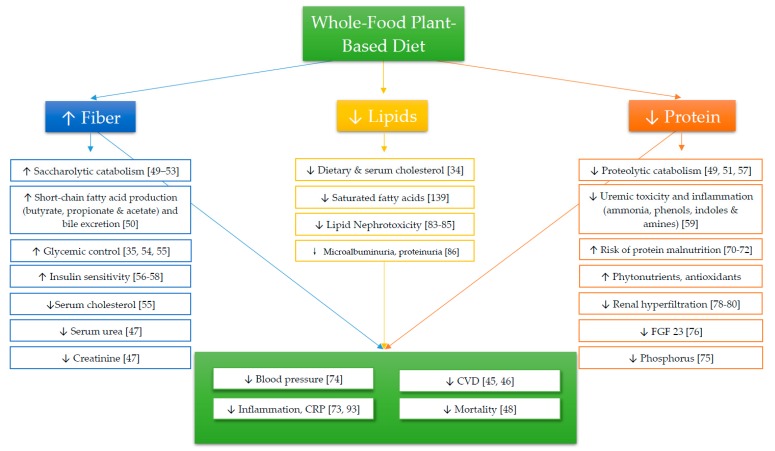
Cited effects of a whole food plant-based diet.

While a majority of individuals diagnosed with CKD undergo pharmaceutical treatments, many of the prescribed drugs relating to this disease and its comorbidities have negative health consequences [29,30] and do not address the behavioral and environmental problems associated with CKD. Therefore, it is imperative to establish an intervention that decreases the pill-burden associated with CKD [31], alleviates the comorbidities of CKD and serves as a cost-effective method for preventing end-stage renal disease (ESRD). The goal of this review is to summarize the current evidence for a WFPB dietary intervention and give an overview of how comorbid conditions of CKD, ESRD and mortality rates can be ameliorated with a WFPB diet (Figure 1). By describing components of a WFPB diet (i.e., macronutrient and micronutrient content), this review will report unequivocal evidence for the utility of a whole food plant-based diet in the prevention and treatment of CKD. Additionally, we will discuss the potential negative side effects of a WFPB diet and limitations to its utility in individuals with CKD.

## 2. Carbohydrates

It is recommended that individuals with CKD get at least 60% of their total energy intake from CHO [32]. A WFPB diet is typically high in CHO, as established in previous dietary interventions. Models similar to a WFPB diet provide roughly 75% of daily energy from CHO sources [33,34]. However, it has been established that approximately 44% of patients develop CKD due to pre-existing diabetes, making glycemic control a major issue in this population [3]. A study by Anderson et al. demonstrated that a high-CHO, high-fiber diet can improve glycemic control in individuals with insulin-treated diabetes [35].

### Fiber

Fiber is a type of indigestible CHO that is readily available in plant foods and which enters the digestive system via the consumption of plants or plant-based products. Fiber has numerous physiological functions, many of which have been reported to provide health benefits and prevent chronic diseases [36,37,38,39,40,41]. The average American ingests 16 grams of fiber each day, far less than the recommended 28 and 34 grams—suggested by the U.S. Department of Health and Human Services (HHS) and U.S. Department of Agriculture (USDA)—for women and men, respectively [42,43]. There is well documented literature demonstrating CVD reduction with increased fiber intake, which is vital to CKD patients because the majority of CKD deaths are ultimately caused by CVD rather than ESRD [44,45,46]. In addition to conferring cardiovascular benefits, a median fiber intake of ~27 g/day reduces serum urea and creatinine levels in CKD patients, which are common markers of an abnormal glomerular filtration rate (GFR) [47]. In a study of NHANES III data conducted by Krishnamurthy et al. [48], it was found that a higher total and insoluble fiber intake attenuated the mortality rate, whereas soluble fiber had no effect on mortality. The mechanism behind the relationship of fiber with chronic disease is thought to be largely attributable to the gut microbiota, where higher fiber intake promotes fermentation and the saccharolytic (i.e., action by bacteria that primarily ferment CHO) catabolism of food, resulting in a downstream increase in beneficial short chain fatty acid (e.g., butyrate, propionate and acetate) production and bile excretion [49,50,51,52,53]. This upregulation serves to decrease serum cholesterol and glucose absorption while simultaneously increasing insulin sensitivity [54,55,56,57,58]. With increased fiber intake, the gut microbiota shifts, increasing the quantity of microflora that process and break down fiber-rich foods. This microflora also traps protein nitrogen, resulting in increased nitrogen excretion, and therefore decreased urea in the bloodstream [47]. Additionally, fiber decreases the amount of time for protein fermentation in the intestinal tract, resulting in decreased bacterial metabolites such as ammonia, phenols, indoles and amines derived from proteolytic (i.e., action by bacteria that primarily ferment protein) catabolism that contribute to uremic toxicity and cause an inflammatory state [49,51,59]. Reduced protein intake could slow the buildup of uremic toxins in the colon, mainly by replacing dietary protein with a plant-based food that contains fiber. It should be noted that fiber also plays several roles in satiety, food digestion and excretion. Fiber tends to make individuals feel fuller longer because it delays gastric emptying and promotes satiating gut hormones [57,60], which decrease the amount of food that people consume and the frequency with which they consume them. Insoluble fiber provides bulk and promotes regular movement through the gastrointestinal tract, which is beneficial in both the CKD and ESRD populations, which often experience slower colonic transit times [61]. Additionally, fiber has been shown to decrease fasting blood glucose in individuals with TIIDM, which is a major cause of CKD [3,62]. Soluble fiber works to decrease the glycemic load of CHO by slowing the digestion rate of foods, which in turn attenuates the insulin response. Vegan and/or WFPB diets tend to be significantly higher in fiber than normal diets or low protein diets, which indicates that adopting this way of eating could provide many benefits from fiber alone [51].

## 3. Protein

Although high protein diets have been shown to be beneficial in sustaining weight loss [63,64], it is recommended that CKD patients adhere to a low-protein (0.6–0.8 g/kg/day) diet or a very low-protein (0.4–0.3 g/kg/day) diet to reduce uric acid buildup and nephrotoxicity [32,65,66,67]. Results from the Modification of Diet in Renal Disease (MDRD) suggest that a low protein diet may provide a small benefit for individuals with renal insufficiency, but it does not ameliorate the progression of nephropathy in CKD patients [21] and it risks protein malnutrition [68,69]. Since there is already increased protein wasting and a risk of cachexia (wasting syndrome) associated with this population, a large reduction in consumption of protein may be detrimental to CKD patients [70,71,72]. Rather than recommending decreased protein intake, it may be beneficial to change the protein source, as type may be more important than quantity [23,73]. While the structure of amino acids derived from plants is not physically different than of those derived from animals, plant proteins are typically ingested alongside other plant products such as fiber, phytonutrients and antioxidants, whereas animal proteins are typically ingested alongside saturated fats and cholesterol. Consequently, plant-based protein has been associated with greater decreases in blood pressure than animal protein [74]. Additionally, animal-based protein has been shown to increase blood levels of phosphate and fibroblast growth factor 23 (FGF23), which upregulates in order to compensate for high phosphate levels as nephron function declines [75,76]. Animal protein also promotes proteolytic fermentation and subsequently results in increased indoles and phenols in the intestinal tract, decreased insulin sensitivity and increased oxidative stress [49,51,59,77]. Specific to the kidneys, animal-based protein induces hyperfiltration [78], a state that temporarily overworks the kidneys, while an equivalent amount of plant protein does not induce the same stress [79,80]. Occasional hyperfiltration is not harmful, but the regular intake of animal proteins over many years can stress this system, resulting in declining kidney function as we age [28].

## 4. Lipids

It is recommended that individuals diagnosed with CKD get up to 30% of their total energy intake from fat sources [32]. Although an ad libitum WFPB diet is not restrictive of lipid intake, it recommends a decreased consumption of processed oils and saturated fat. Previous studies of vegan and WFPB diets have limited fat intake to comprise less than 15% of daily caloric energy [33,34].

### 4.1. Cholesterol

The highly vascular nature of kidneys implies that they can be affected just as readily, if not more so, by plaque buildup as the heart [81,82]. Lipid nephrotoxicity is a theory that glomerulosclerosis develops in the capillary beds of the kidneys just as atherosclerosis develops in the heart, which was confirmed in 1955 by Hartroft et al. in an analysis of post-mortem kidney autopsies [83,84,85]. In a study by Lin et al. [86], it was found that animal protein, animal fat and cholesterol were all associated with increased microalbuminuria and proteinuria, which are markers of decreased kidney function. In the same study, vegetable intake was identified to have a slight inverse relationship with microalbuminuria, indicating no association between plant-based protein and decline of kidney function [86]. Recently, medicinal efforts to combat lipid nephrotoxicity include statin drugs to lower cholesterol and slow the progression of CKD [83]. While the theory behind statin use in CKD patients is sound, dietary change can be a more cost effective and sustainable option to reduce cholesterol levels. A study by Barnard et al. conducted a 74-week intervention in individuals with TIIDM and found that total cholesterol was decreased by 20.4 mg/dL on a low-fat vegan diet and 6.8 mg/dL on a diet following the 2003 American Diabetes Association (ADA) guidelines [34]. In the same study, LDL cholesterol was reduced in both the vegan (∆ −13.5 mg/dL) and ADA (∆ −3.4 mg/dL) conditions, but a greater reduction was found in the group that was prescribed the vegan diet.

### 4.2. Omega-3 Fatty Acids

Omega-3 fatty acids are known to reduce inflammation [87], serum triglycerides, blood pressure and heart rate while increasing serum HDL cholesterol [88]. Plant-derived omega-3 fatty acids, known as α-linoleic acids, are commonly found in vegetable oils, as well as in foods such as flaxseeds, chia seeds, olives, walnuts, broccoli, brussels sprouts and soybeans. The ingestion of 1.5 to 3 g/day of α-linoleic acid has shown utility in preventing the development of CVD in patients with CKD [89]. In a meta-analysis of randomized controlled trials that administered (both fish- and plant-derived) omega-3 fatty acid supplements, the risks of myocardial infarction and all-cause mortality were both reduced by 20% compared to control or placebo [90]. Additionally, plant-based omega-3 fatty acids may be beneficial for individuals who progress to ESRD and require dialysis. In a study by Mirfatahi et al., high doses of flaxseed oil (6 g/day) were found to attenuate markers of bone reabsorption by up to 17% in hemodialysis patients [91]. Although the effects of consuming foods rich in α-linoleic acid may be more modest than those of eicosapentaenoic acid (EPA) [92], there is still a benefit to consuming plant-based omega-3 fatty acids to reduce CVD, comorbid disease and all-cause mortality in CKD and/or ESRD patients.

## 5. Inflammation

C-reactive protein (CRP) is one of the most frequently used biomarkers for measuring acute and chronic systemic inflammation. Blood levels of CRP can change in as little as 24 hours in response to acute inflammation, and it is one of the strongest predictors of future CVD [93]. Saturated fat has been found to have a slight positive relationship with CRP levels, indicating that it may be linked to disease states related to inflammation [94]. Conversely, many foods and food products have been shown to decrease inflammation, acid load and mortality in individuals with a GFR of < 60 mL/min/1.73 m^2^ [75,95]. Previous research has identified plant foods such as cherries [96], fiber [48,97] and whole grains [98] to have inverse relationships with CRP. Soy, in particular, represents a complete plant protein that may have anti-inflammatory and renoprotective properties [95,99]. In a study by Azadbakht et al. [100], CRP levels significantly decreased in individuals who consumed soy-based protein for four years as compared to those who consumed animal protein during the same amount of time. Others have shown no difference in inflammatory markers among soy-consuming participants [101,102], but have noted an inverse relationship between CRP and serum isoflavone, which is a phytoestrogen—or a plant-based compound—with estrogen-like qualities that is derived from soy [103]. Foods with anti-inflammatory properties may be beneficial in ameliorating CKD, but more research needs to be done to further strengthen the association.

## 6. Acidity and Alkalinity of Foods

Acid-base balance is maintained by harmonizing non-carbonic acids (e.g., sulfuric acid and phosphoric acid) and bases (e.g., bicarbonate). Acid in the diet comes primarily from protein, which, when metabolized, releases sulfuric acid, whereas bases such as bicarbonate are metabolized from the organic anion salts found in fruits and vegetables [14,104,105]. Metabolic acidosis is a condition in which levels of acidity are high relative to base production. It can result from a prolonged elevated acid load due to high endogenous acid production, the inability of the kidneys to excrete acid, or a deficiency of bicarbonate, which serves as an acid buffer [11,14]. A study by Goraya et al. [106] aimed to treat metabolic acidosis in stage 4 CKD patients, by comparing the effects on acid load of the administration of a sodium bicarbonate (i.e., baking soda) supplement with those of a fruit and vegetable supplement over one year. Sodium bicarbonate administration had a similar effect to fruit and vegetable intake on acid state, but it was noted that sodium chloride, a byproduct of the reaction between sodium bicarbonate and hydrochloric acid (HCl) in the stomach, increased upon the consumption of sodium bicarbonate. Sodium chloride (NaCl), commonly known as table salt, further promotes acidosis and tends to be a negative predictor of bicarbonate levels in the blood [107]. Although the study by Goraya et al. [106] shows potential for reducing acid load via a sodium bicarbonate supplement, it may not be a sustainable option since it promotes higher amounts of the non-metabolized ion chloride [107,108]. The fruit and vegetable condition, however, both improved metabolic acidosis and decreased systolic blood pressure (BP) without generating hypernatremia or hyperkalemia, indicating that increasing fruit and vegetable intake is a sustainable option for reducing dietary acid load [106].

While the typical Western diet promotes a higher acid load, with its heavy intake of animal-based products, fruit and vegetable intake has been shown to reduce metabolic acidosis in CKD patients [109]. Dietary acid is commonly measured by calculating a score for foods and adding them together for the total acid load. The potential renal acid load (PRAL) score of foods is used as an indicator of dietary acid-base load, which is then assessed for CKD patients and used as a guide when adopting a low-acid diet. The calculation for the PRAL score takes into account dietary protein, phosphorus, potassium, calcium and magnesium, and is calculated using the following formula [110,111]:

Potential renal acid load = 0.4888 × dietary protein (g) + 0.0366 × dietary phosphorus (mg) − 0.0205 × dietary potassium (mg) − 0.0125 × calcium (mg) − 0.0263 × magnesium (mg)(1)

Positive PRAL values indicate acid-containing foods, whereas negative PRAL values indicate alkaline foods. Common foods and their PRAL values can be found in Table 2. On average, plant-based foods have a lower PRAL score than animal-based foods and, when consumed, can decrease serum creatinine levels and therefore slow CKD progression [10,112,113]. Lower PRAL scored foods are recommended to CKD patients in order to prevent metabolic acidosis. A diet promoting a low PRAL score can be used to predict and improve the acidity of the urine in a relatively short time frame [110]. In a study by Cosgrove et al. [114], subjects who were administered a low PRAL, vegan (i.e., plant-based) diet for only 7 days had improved urine pH and lowered dietary cholesterol.

## 7. Electrolyte Balance

A common concern with increased plant intake is the overconsumption or over-accumulation of minerals that are contraindicated in CKD patients, specifically in those with late-stage (stages IV and V) CKD (eGFR ≤ 29 mL/min/1.73 m^2^). The minerals of greatest concern include potassium, phosphorus and sodium. These minerals tend to be in higher abundance in plant foods, but findings indicate that there is less absorption of naturally-found minerals from plant foods than there is from animal sources. The effect of a WFPB diet on the electrolyte balance in mid-spectrum and late-stage CKD remains unknown.

### 7.1. Hyperkalemia

Late-stage CKD patients are at risk for hyperkalemia, or an overabundance of potassium (K^+^) in the blood (> 5.1 mEq/L), which can cause an irregular or abnormal heartbeat and put patients at greater risk for a cardiac event [115]. Generally, when a patient is hyperkalemic, physicians may recommend a potassium binding drug such as Kayexalate and/or the restriction of potassium-rich foods such as potatoes, avocados, strawberries, bananas, spinach and oranges. Although there is typically a greater amount of potassium found in plant-based foods, there tends to be less bioavailability in these foods as compared to in animal-based or more heavily processed food products. Additionally, the potassium in fruits and vegetables is typically ingested with the non-chloride anion, bicarbonate, that promotes increased potassium excretion via the urine [116]. Although previous studies have demonstrated that increasing fruit and vegetable intake does not induce hyperkalemia in CKD patients [106,109], the effect of a WFPB diet on late-stage CKD is unknown. Further research needs to be conducted on the risk of hyperkalemia in individuals with CKD who consume a WFPB diet or a vegan diet.

### 7.2. Hyperphosphatemia

The protein FGF23 is responsible for excreting phosphate and decreasing its reabsorption. When the kidneys are compromised during renal failure, they are unable to excrete phosphate, creating a state known as hyperphosphatemia (blood phosphate > 4.5 mg/dL), which promotes a compensatory response that drastically upregulates levels of circulatory FGF23 [117]. Blood levels of FGF23 are typically elevated in the CKD population, and can increase beyond 100-fold in advanced CKD [118]. The upregulation of FGF23 in response to high levels of phosphate is similar to the upregulation of insulin in response to excess glucose. In normal, healthy individuals, FGF23 is sufficient to regulate phosphate, but when burdened by an abundance of phosphate, the upregulation of FGF23 to clear it goes relatively unanswered, and circulating levels of both phosphate and FGF23 continue to increase. Ultimately, high levels of phosphate and FGF23 result in negative vascular effects such as vascular calcification, which increases the risk of mortality [119].

Animal protein is the main source of bioavailable phosphorus, as phosphorus is often bound to proteins in vivo. There is a strong positive correlation between animal protein intake and phosphate intake, which is one reason why individuals with CKD are recommended a low protein and/or low phosphate diet. The dietary reference intake suggests that phosphate intake be limited to < 700 mg/day for all adults aged 19 years and older, and the 2019 KDIGO nutrient guidelines report that there is an absence of data that dietary restrictions improve CKD outcomes in stages 3a-4 [43,120]. A low phosphate diet generally restricts foods like poultry, fish, dairy, nuts, soft drinks and oatmeal. Furthermore, some patients are prescribed phosphorus-binding drugs to take with meals such as calcium carbonate, calcium acetate (PhosLo) or Sevelamer, which all work similarly to precipitate phosphate in the gastrointestinal tract, so that it can later be excreted in the stool, rather than being absorbed by the intestine [121,122]. Phosphorus from plant foods proves to be less bioavailable due to the fact that it is primarily bound to phytate, which is poorly absorbed in the gastrointestinal tract, since humans lack the phytase enzyme that breaks down phytate in the gut [75,119,121,123,124]. A WFPB diet could prove beneficial in reducing the phosphorus load on the body, thereby improving vascular effects and potentially mediating the effects of low calcium levels as discussed in the following section.

### 7.3. Hypocalcemia

In individuals with CKD, blood calcium levels tend to decrease due to high levels of phosphate in the blood, causing hypocalcemia, or a blood calcium level below 8.6 mg/dL. The most recent KDIGO guidelines recommend that calcium binders be restricted in all stages of CKD [120]. Due to nephron damage, the kidneys are unable to effectively excrete electrolytes, causing an increase in blood phosphate that binds to calcium to form calcium phosphate, which subsequently decreases available calcium. The resulting low calcium level stimulates the parathyroid gland to produce parathyroid hormone (PTH), which causes the bones to release calcium in an effort to increase the serum calcium level. Additionally, the kidneys play a crucial role in converting the vitamin D that is absorbed from food or through sun exposure to its active form, but when the kidneys are in a diseased state, the ability to convert 25-(OH) vitamin D into the active form, 1,25 dihydroxy-vitamin D, is reduced [125]. This has implications for the maintenance of calcium and phosphate homeostasis, because in the absence of vitamin D, only 10–15% of dietary calcium and 60% of phosphate is absorbed [126,127,128].

### 7.4. Hypernatremia

Hypernatremia, or a high level of sodium in the blood, is an issue for both CKD and ESRD patients, as it can increase total blood volume and therefore blood pressure, resulting in HTN. Individuals with CKD are recommended to reduce sodium intake below 2.3 g/day (equivalent to 6 grams of NaCl or table salt), but this can be challenging because sodium intake may be a result of the typical Western diet [32]. Additional sodium can also be introduced into the digestive system by attempting to correct metabolic acidosis through the intake of sodium bicarbonate [106]. In addition to contributing to HTN, sodium chloride has been shown to produce metabolic acidosis, which is often a comorbidity and a contributor to CKD [107,129].

## 8. Potential Drawbacks to a Plant-Based Diet

### 8.1. Vitamins and Minerals

Plant-based diets typically must be supplemented with vitamin B12, which is a vitamin that is traditionally ingested by drinking unfiltered water or eating unclean plant foods. Current standards of water purification minimize the amounts of B12 in the water, and fruit and vegetables are typically washed of any dirt or soil remnants before consumption. An individual relying on an animal-based diet receives B12 through the intake of animal proteins, due to the nature of animals ingesting B12 with their food—which is typically eaten off the ground—and via their water supply, which in many cases is an unpurified reservoir. Additionally, there is concern that a plant-based diet may be deficient in, or interfere with, the absorption of calcium, zinc and iron. Nevertheless, the levels of these micronutrients have been reported to be sufficient in previous studies of plant-based diets, which did not demonstrate that reduced bioavailability has biological consequences [130,131,132,133,134]. However, more research is still needed to carefully assess the potential for deficiencies in a WFPB diet in a clinical population with CKD.

### 8.2. Calorie Consumption

The current RDA for daily caloric intake in individuals with CKD is 30–35 kcal/kg/day. Many individuals who switch to a WFPB diet have difficulty consuming an adequate number of calories. It is important to include a variety of plant-based products such as starchy vegetables and fruits to achieve energy balance, as it is very difficult to get energy from foods that have little caloric value (e.g., leafy greens). High amounts of fiber, when introduced rapidly, can make individuals feel full and can create discomfort in the gastrointestinal tract, which can further deter them from achieving adequate caloric intake.

### 8.3. Accessibility

In Western societies, a WFPB diet can be seen as counter-cultural and is difficult to adhere to. Approximately 5% of people in the U.S. consider themselves to be vegetarian, and even fewer (2%) consider themselves vegan, or their diets fully plant-based [135]. Not only is there a large social barrier when switching to a plant-based diet, but there are also barriers in terms of the availability and palatability of plant-based foods [136]. In a study by Banerjee et al. [137], it was found that 4.5% of CKD patients are food insecure, which independently increases the chance of developing ESRD. Limited access to foods, specifically fresh fruits and vegetables, as well as unprocessed food products can present a unique challenge to anyone trying to adhere to a WFPB diet. Additionally, the WFPB diet can require extra preparation in order to avoid heavy processing, and the lack of time and skills to prepare WFPB meals can further deter individuals from adopting this lifestyle.

## 9. Conclusions

Current RDAs for CKD patients suggest following a diet with low protein (0.3–0.8 g/kg/day), sodium (<2.3 g/day), phosphate (<700 mg/day) and the supplementation of essential amino acids, ketoacids, calcium carbonate, vitamins and iron [32,138]. This dietary approach suggests that patients achieve approximately 60% of total energy intake from CHO, 30% from fat and 10% from protein. Previous studies administering a low-fat vegan diet generally prescribed diets that consisted of 75% energy intake from CHO, 10% from fat and 15% from protein [33,34]. Although these diets differ slightly in macronutrient content, this may be less important than the overall nutritional value and digestion process of the foods consumed. The introduction of a diet that is high in fiber, vitamins and minerals has implications for slowing the progression of CKD. This lifestyle change has the potential to prevent premature death, improve quality of life and mitigate comorbid diseases, specifically CVD, which is the leading cause of mortality in CKD patients in the U.S. Through adherence to a WFPB diet or by simply increasing fruit and vegetable intake, CKD may be attenuated or prevented if identified early. By adopting the habits associated with a WFPB diet, many of the chronic disease outcomes could be reduced. A WFPB diet is a viable alternative to current dietary recommendations for CKD patients; however, further research is needed to determine the effects that a long-term WFPB diet has on the progression of CKD and the feasibility of this dietary lifestyle change.

## 10. Literature Search

This review was accomplished by searching PubMed and Google Scholar for scientific articles containing information on whole food plant-based diets and chronic kidney disease using the key words “whole food”, “plant-based”, “vegan”, “vegetarian”, “chronic kidney disease”, “CKD” and “renal failure”. Additional articles were identified for other clinically-relevant topics (e.g., cardiovascular disease, diabetes risk and inflammation) and were discussed in subsequent sections of the review article.

## Figures and Tables

**Table 1:** The stages of chronic kidney disease (CKD) by glomerular filtration rate (GFR) category.

GFR Category	Description	Estimated GFR (mL/min/1.73 m^2^)
Stage 1	Normal or high	> 90
Stage 2	Mildly decreased	60–89
Stage 3a	Mildly to moderately decreased	45–59
Stage 3b	Moderately to severely decreased	30–44
Stage 4	Severely decreased	15–29
Stage 5	Kidney Failure	<15

The glomerular filtration rate must be persistent for 3 or more months in order to classify the stage. This table was adapted from the 2012 Kidney Disease Improving Global Outcomes (KDIGO) CKD Guideline.

**Table 2:** Common foods and their potential renal acid load (PRAL) values. Each score is calculated based on a 100 g portion. Positive PRAL values (red) indicate acid-containing foods whereas negative PRAL values (green) indicate alkaline foods. Table adapted from.

Category	Food	PRAL (per 100 g)
**Fruits**	Apples	−1.9
	Bananas	−6.9
	Lemons	−2.3
	Medjool Dates	−13.7
	Oranges	−3.6
**Vegetables**	Broccoli	−4.0
	Carrots	−5.7
	Kale	−8.3
	Spinach	−11.8
	Sweet Potato	−5.6
**Grains**	Brown Rice	7.5
	Oats	13.3
	Spaghetti (white)	7.3
	White Bread	6.0
**Legumes**	Garbanzo Beans (Chickpeas)	0.3
	Kidney Beans	−8.4
	Lentils	5.4
	Peanuts	6.2
	Soybeans	−4.7
	Tofu (raw)	−0.3
	White Beans	−23.2
**Nuts and Seeds**	Almonds	2.3
	Flaxseeds	2.1
	Pecans	2.1
	Sunflower Seeds	12.1
**Fats**	Butter	0.6
	Corn Oil	0
	Margarine	−0.5
	Olive Oil	0
**Meats**	Beef (Lean)	8.7
	Chicken	13.2
	Fish (average)	7.9
	Turkey	9.0
**Dairy and Eggs**	Cottage Cheese	23.4
	Egg (Whole)	0.6
	Hard cheese	34.2
	Ice Cream	28.7
	Whole Milk	1.2
	Whole Milk Yogurt	1.5
